# Supplementary material for: Nicking mechanism underlying the DNA phosphorothioate-sensing antiphage defense by SspE
Source: Nat Commun. 2022 Nov 9;13:6773. doi: 10.1038/s41467-022-34505-0 (PMC9646914; doi:10.1038/s41467-022-34505-0)
Supplement: Supplementary file 1 — Supplementary Information [file 41467_2022_34505_MOESM1_ESM.pdf]

Supplementary Information

Nicking mechanism underlying the DNA phosphorothioate-sensing antiphage defense by SspE

Haiyan Gao[1,2#], Xinqi Gong[3,#], Jinchuan Zhou[1], Yubing Zhang[1,2], Jinsong Duan[4], Yue Wei[1,5], Liuqing Chen[2], Zixin Deng[1], Jiawei Wang[4], Shi Chen[1,5*], Geng Wu[2*], Lianrong Wang[1*]

Supplementary Table 1. Data collection and refinement statistics of $SspE_{CTD}$ from *S. scabiei* DSM 41658

	**Native $SspE_{CTD}$**	**Se-Met $SspE_{CTD}$**
**Data collection**		
Space group	$C222_1$	$C222_1$
Cell dimensions		
a, *b*, *c* (Å)	108.7, 278.9, 182.3	107.9, 277.7, 181.8
α, β, γ (°)	90, 90, 90	90, 90, 90
Wavelength	0.97849 Å	0.97893 Å
Resolution (Å)	50.00-2.73 (2.78-2.73)	50.00-3.00 (3.05-3.00)
R_{merge}	0.116 (0.738)	0.158 (1.189)
CC1/2	0.991 (0.912)	0.994 (0.804)
$I/\sigma I$	26.00 (2.071)	16.00 (1.875)
Completeness (%)	99.8 (97.7)	100.0 (100.0)
Redundancy	13.1 (11.2)	13.2 (12.1)
**Refinement**		
Resolution (Å)	139.46-2.72	
No. reflections	70397	
R_{work}/R_{free}	21.22%/26.50%	
No. atoms		
Protein	14150	
Water	0	
B-factors		
Protein	81.246	
R.m.s. deviations		
Bond lengths (Å)	0.0083	
Bond angles (°)	1.3404	
Ramachandran plot statistics (%)		
Most favorable	95.5	
Additionally allowed	4.0	
Disallowed	0.5	

Only one crystal was used to identify the structure of $SspE_{CTD}$ from *S. scabiei* DSM 41658. Values in parentheses are for the highest-resolution shell.

Supplementary Table 2. Data collection and refinement statistics of SspE from *S. yokosukanensis* DSM 40224

	Full-length SspE
**Data collection**	
Space group	$P2_12_12_1$
Cell dimensions	
a, *b*, *c* (Å)	109.28, 137.82, 292.32
α, β, γ (°)	90, 90, 90
Wavelength	0.97849 Å
Resolution (Å)	50.00-3.30 (3.42-3.30)
R_{merge}	0.259 (1.089)
CC1/2	0.719 (0.779)
$I/\sigma I$	12.4 (1.9)
Completeness (%)	91.4 (93.6)
Redundancy	13.3 (13.6)
**Refinement**	
Resolution (Å)	46.14-3.42
No. reflections	61769
R_{work}/R_{free}	20.1%/27.6%
No. atoms	
Protein	24344
Water	0
B-factors	
Protein	94.2
Water	
R.m.s. deviations	
Bond lengths (Å)	0.014
Bond angles (°)	1.707
Ramachandran plot statistics (%)	
Most favorable	89.5
Additionally allowed	9.7
Disallowed	0.8

Only one crystal was used to identify the structure of SspE from *S. yokosukanensis* DSM 40224. Values in parentheses are for the highest-resolution shell.

Supplementary Table 3. Strains, plasmids, and phages used in this study

Strains and plasmids	Characteristics	Source or reference
**Strains**		
S. yokosukanensis DSM 40224	5'-C_{PS}CS-3' modification, GenBank: LMWN01000000	DSMZ
S. scabiei DSM 41658	5'-C_{PS}CS-3' modification, GenBank: NZ_LBNJ01000000	DSMZ
E. coli BL21(DE3)	F^- *ompT gal dcm lon hsdS*$_B$($r_B^- m_B^-$) λ(DE3 [*lacI lacUV5*-T7p07 *ind*1 *sam*7 *nin*5]) [*mal*B^+]$_{K-12}$($λ^S$)	Novagen
S. lividans HXY6	*S. lividans* 1326 derivative lacking *dnd* and *ssp* genes	[1]
**Phages**		
JXY1	*Podoviridae*, lytic, dsDNA	[2]
**Plasmids**		
pBluescript Ⅱ SK(+)	Cloning vector, 3 kb, Amp^r	[3]
pUC19	Cloning vector, 2.7 kb, Amp^r	TransGen Biotech
pSET152	*E. coli*-*Streptomyces* shuttle vector, *aac(3)*IV, ColEI, *att*$^{\phi C31}$, *ori* T	[4]
pPT551	pET28a derivative expressing the CTD of SspE from *S. scabiei* DSM 41658, expression vector	This work
pPT552	pET28a derivative expressing the NTD of SspE from *S. yokosukanensis* DSM 4022*4*, expression vector	This work
pPT553	pET28a derivative expressing the CTD of SspE from *S. yokosukanensis* DSM 40224, expression vector	This work
pPT555	pET28a derivative expressing $SspE_{K40A}$ from *S. yokosukanensis* DSM 40224, expression vector	This work
pPT556	pET28a derivative expressing $SspE_{Q31A}$ from *S. yokosukanensis* DSM 40224, expression vector	This work
pPT557	pET28a derivative expressing $SspE_{Y30A}$ from *S. yokosukanensis* DSM 40224, expression vector	This work
pWHU3643	pET28a derivative expressing $SspE_{R100A}$ from *S. yokosukanensis* DSM 40224, expression vector	[2]
pPT558	pET28a derivative expressing $SspE_{R404A/R408A}$ from *S. yokosukanensis* DSM 40224, expression vector	This work
pWHU3261	pET28a derivative expressing $SspE_{H639A}$ from *S. yokosukanensis* DSM 40224, expression vector	[2]
pPT560	pET28a derivative expressing $SspE_{N667A}$ from *S.*	This work

	yokosukanensis DSM 40224, expression vector	
pPT561	pET28a derivative expressing $SspE_{N676A}$ from *S. yokosukanensis* DSM 40224, expression vector	This work
pPT562	pET28a derivative expressing CFP, expression vector	This work
pPT563	pET28a derivative expressing YFP-SspE-CFP, expression vector	This work
pPT564	pET28a derivative expressing YFP-CFP, expression vector	This work
pPT565	pET28a derivative expressing $CTD_{R404A/R408A}$ from *S. yokosukanensis* DSM 40224, expression vector	This work
pPT566	pET28a derivative expressing CTD_{R512A} from *S. yokosukanensis* DSM 40224, expression vector	This work
pPT567	pET28a derivative expressing $CTD_{K395A/R526A}$ from *S. yokosukanensis* DSM 40224, expression vector	This work
pPT568	pET28a derivative expressing $CTD_{396\text{-}771aa}$ from *S. yokosukanensis* DSM 40224, expression vector	This work
pPT569	pET28a derivative expressing $CTD_{441\text{-}771aa}$ from *S. yokosukanensis* DSM 40224, expression vector	This work
pWHU3658	pSET52 derivative with a 9.7-kb fragment carrying *sspABCDE* from *S. yokosukanensis* DSM 40224	[2]
pPT581	pWHU3658 derivative expressing $SspABCDE_{K40A}$	This work
pPT582	pWHU3658 derivative expressing $SspABCDE_{R404A/R408A}$	This work
pPT583	pWHU3658 derivative expressing $SspABCDE_{N667A}$	This work
pPT584	pWHU3658 derivative expressing $SspABCDE_{N676A}$	This work
pPT585	pWHU3658 derivative expressing $SspABCDE_{Q31A}$	This work
pPT586	pWHU3658 derivative expressing $SspABCDE_{Y30A}$	This work
pPT590	pSET152 derivative with a 2.3-kb fragment carrying *sspE* from *S. yokosukanensis* DSM 40224	This work
pPT592	pPT590 derivative expressing $SspE_{K40A}$	This work
pPT593	pPT590 derivative expressing $SspE_{R404A/R408A}$	This work
pPT594	pPT590 derivative expressing $SspE_{N667A}$	This work
pPT595	pPT590 derivative expressing $SspE_{N676A}$	This work
pPT596	pPT590 derivative expressing $SspE_{Q31A}$	This work
pPT597	pPT590 derivative expressing $SspE_{Y30A}$	This work

Supplementary Table 4. Data collection and refinement statistics of $SspE_{R100A}$ from *S. yokosukanensis* DSM 40224

	$SspE_{R100A}$
**Data collection**	
Space group	$P2_12_12_1$
Cell dimensions	
a, *b*, *c* (Å)	110.03, 137.97, 292.35
α, β, γ (°)	90, 90, 90
Wavelength	0.97849 Å
Resolution (Å)	50.00-3.50 (3.56-3.50)
R_{merge}	0.306 (2.296)
CC1/2	0.988 (0.583)
$I/\sigma I$	15.0 (2.0)
Completeness (%)	99.7 (85.8)
Redundancy	12.9 (11.5)
**Refinement**	
Resolution (Å)	36.54-3.48
No. reflections	54087
R_{work}/R_{free}	25.7%/30.8%
No. atoms	
Protein	24462
Water	0
B-factors	
Protein	49.7
Water	
R.m.s. deviations	
Bond lengths (Å)	0.016
Bond angles (°)	1.829
Ramachandran plot statistics (%)	
Most favorable	87.4
Additionally allowed	11.9
Disallowed	0.7

Only one crystal was used to identify the structure of $SspE_{R100A}$ from *S. yokosukanensis* DSM 40224. Values in parentheses are for the highest-resolution shell.

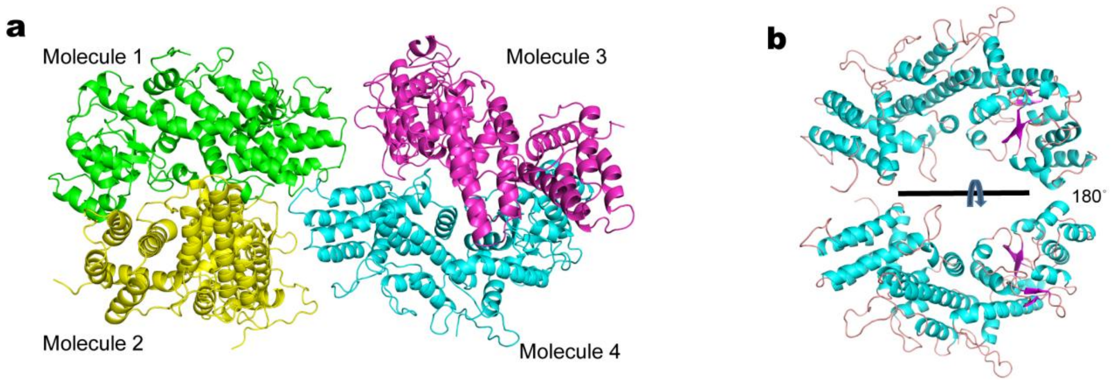

Supplementary Fig. 1. Crystal structures of SspE$_{CTD}$ from *S. scabiei* DSM 41658. **(a)** Ribbon diagram of the four molecules, shown in green, yellow, magenta, and cyan, in one asymmetric unit of SspE$_{CTD}$. (**b**) Rotation (180°) of the SspE$_{CTD}$ monomer along the *x*-axis to show the back view. Helices are shown in cyan, and sheets are shown in magenta. Source data are provided as a Source Data file.

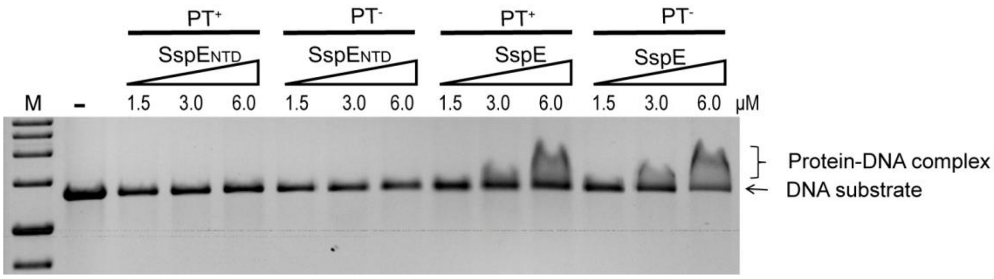

Supplementary Fig. 2. EMSA analysis of the binding of $SspE_{NTD}$ with increasing concentrations to linearized pUC19 DNA with or without PT modification at 5'-C_{PS}CA-3'. The binding interaction between SspE and pUC19 DNA was used as a reference. Source data are provided as a Source Data file.

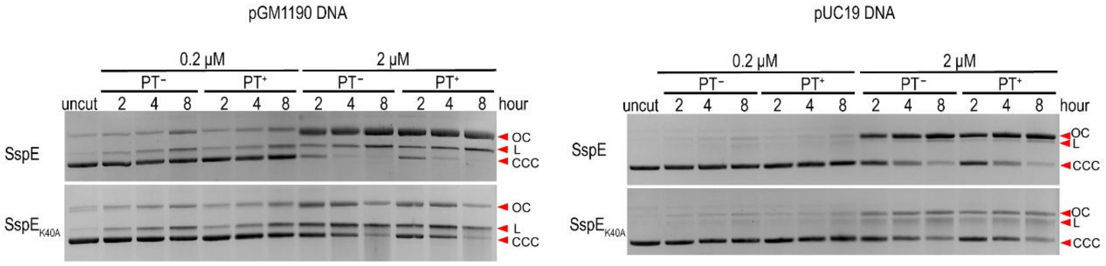

Supplementary Fig. 3. DNA nicking assay of SspE and $SspE_{K40A}$ toward plasmid DNA *in vitro*. In this assay, supercoiled pUC19 and pGM1190 DNA with or without the PT modification were used as substrates. Three hundred nanograms of plasmid DNA were incubated with 0.2 or 2 μM protein at 28 °C in CutSmart buffer (New England Biolabs). At the indicated time points, the nicked DNA products were analyzed on 1% agarose gels. PT-modified pGM1190 and pUC19 were isolated from SspABCD-SspE-expressing *S. lividans* HXY6 and *E. coli* cells, respectively. Source data are provided as a Source Data file.

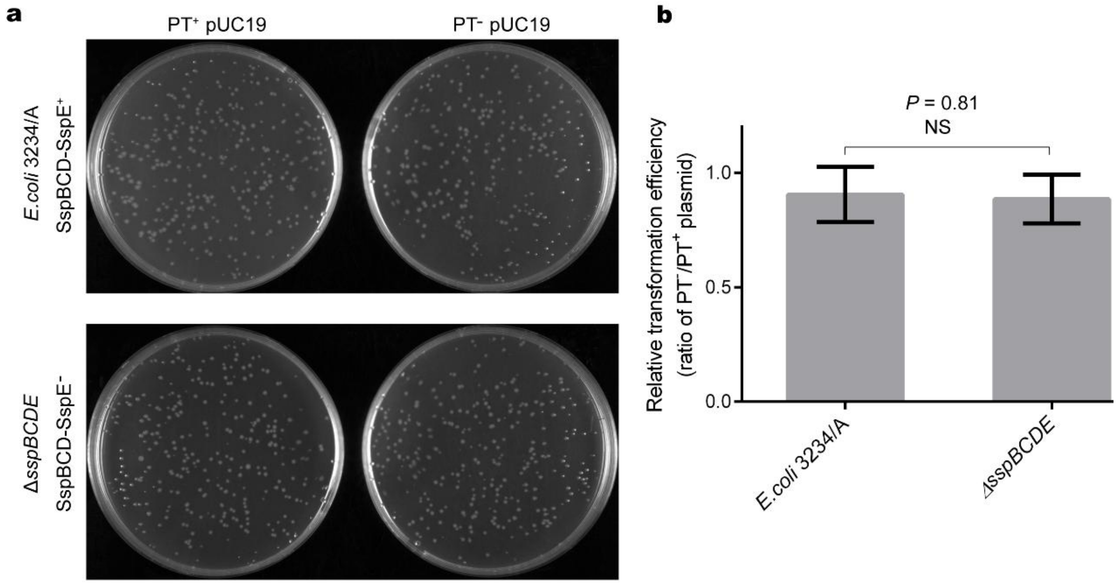

Supplementary Fig. 4. Assessment of the antiplasmid activity of SspE in *E. coli* 3234/A. (a) Wild-type SspABCD-SspE module-containing *E. coli* 3234/A and Ssp-lacking mutant Δ*sspBCDE* were used as host strains for transformation by equal amounts of PT-modified (PT^+) or non-PT-modified (PT^-) pUC19, respectively. **(b)** The results are presented as relative transformation efficiencies (ratios of PT^-/PT^+ plasmid) obtained by parallel transformation of PT^-/PT^+ plasmid DNA. Data and error bars represent the mean ± SD from four independent experiments. Statistical significance was calculated by unpaired two-sided Student t-tests; NS, not significant. Source data are provided as a Source Data file.

Supplementary References

1 Liang, J. *et al.* DNA modification by sulfur: analysis of the sequence recognition specificity surrounding the modification sites. *Nucleic Acids Res.* **35**, 2944-2954 (2007).

2 Xiong, X. *et al.* SspABCD–SspE is a phosphorothioation-sensing bacterial defence system with broad anti-phage activities. *Nat. Microbiol.* **5**, 917-928 (2020).

3 Alting-Mees, M. A. & Short, J. M. pBluescript II: gene mapping vectors. *Nucleic Acids Res.* **17**, 9494 (1989).

4 Bierman, M. *et al.* Plasmid cloning vectors for the conjugal transfer of DNA from Escherichia coli to Streptomyces spp. *Gene* **116**, 43-49 (1992).
