# Supplementary material for: Nicking mechanism underlying the DNA phosphorothioate-sensing antiphage defense by SspE
Source: Nat Commun. 2022 Nov 9;13:6773. doi: 10.1038/s41467-022-34505-0 (PMC9646914; doi:10.1038/s41467-022-34505-0)
Supplement: Supplementary file 3 — Reporting Summary [file 41467_2022_34505_MOESM3_ESM.pdf]

nature portfolio

Corresponding author(s): Lianrong Wang, Geng Wu, Shi Chen

Last updated by author(s): Oct 1, 2022

Reporting Summary

Nature Portfolio wishes to improve the reproducibility of the work that we publish. This form provides structure for consistency and transparency in reporting. For further information on Nature Portfolio policies, see our Editorial Policies and the Editorial Policy Checklist.

Statistics

For all statistical analyses, confirm that the following items are present in the figure legend, table legend, main text, or Methods section.

n/a	Confirmed	
☐	☒	The exact sample size (*n*) for each experimental group/condition, given as a discrete number and unit of measurement
☐	☒	A statement on whether measurements were taken from distinct samples or whether the same sample was measured repeatedly
☐	☒	The statistical test(s) used AND whether they are one- or two-sided<br>*Only common tests should be described solely by name; describe more complex techniques in the Methods section.*
☒	☐	A description of all covariates tested
☒	☐	A description of any assumptions or corrections, such as tests of normality and adjustment for multiple comparisons
☐	☒	A full description of the statistical parameters including central tendency (e.g. means) or other basic estimates (e.g. regression coefficient) AND variation (e.g. standard deviation) or associated estimates of uncertainty (e.g. confidence intervals)
☐	☒	For null hypothesis testing, the test statistic (e.g. *F*, *t*, *r*) with confidence intervals, effect sizes, degrees of freedom and *P* value noted<br>*Give P values as exact values whenever suitable.*
☒	☐	For Bayesian analysis, information on the choice of priors and Markov chain Monte Carlo settings
☒	☐	For hierarchical and complex designs, identification of the appropriate level for tests and full reporting of outcomes
☒	☐	Estimates of effect sizes (e.g. Cohen's *d*, Pearson's *r*), indicating how they were calculated

Our web collection on statistics for biologists contains articles on many of the points above.

Software and code

Policy information about availability of computer code

Data collection	Crystal diffraction data were collected on the BL19U1 beamline at the National Center for Protein Sciences Shanghai (NCPSS, Shanghai, China).
Data analysis	Graph.prism (v6), HoDock, Pymol (v2.4.0), HKL2000, COOT, and CCP4 were used to analysis the search results.<br>The licenses for the above software were described as follow:<br>Graphpad.prism: protected under the copyright law, but FREE for use in research and education.<br>HoDock: protected under the copyright law, but FREE for use in research and education.<br>Pymol: protected under the copyright law, but FREE for use in research and education.<br>HKL2000: protected under the copyright law, but FREE for use in research and education.<br>COOT: protected under the copyright law, but FREE for use in research and education.<br>CCP4: protected under the copyright law, but FREE for use in research and education.

For manuscripts utilizing custom algorithms or software that are central to the research but not yet described in published literature, software must be made available to editors and reviewers. We strongly encourage code deposition in a community repository (e.g. GitHub). See the Nature Portfolio guidelines for submitting code & software for further information.

Data

Policy information about availability of data

All manuscripts must include a data availability statement. This statement should provide the following information, where applicable:

- Accession codes, unique identifiers, or web links for publicly available datasets
- A description of any restrictions on data availability
- For clinical datasets or third party data, please ensure that the statement adheres to our policy

The data supporting the findings of this study are available from the corresponding author on request. The coordinates and structure factors of SspE-CTD from S. scabiei DSM 41658 and full-length SspE and SspE-R100A from S. yokosukanensis DSM 40224 have been deposited in the Protein Data Bank under accession numbers 7DRI, 7DRS, and 7DRR, respectively.

Human research participants

Policy information about studies involving human research participants and Sex and Gender in Research.

Reporting on sex and gender	This research didn't involve human research participants.
Population characteristics	This research didn't involve human research participants.
Recruitment	No participants were recruited.
Ethics oversight	This research didn't involve human research participants.

Note that full information on the approval of the study protocol must also be provided in the manuscript.

Field-specific reporting

Please select the one below that is the best fit for your research. If you are not sure, read the appropriate sections before making your selection.

☒ Life sciences ☐ Behavioural & social sciences ☐ Ecological, evolutionary & environmental sciences

For a reference copy of the document with all sections, see nature.com/documents/nr-reporting-summary-flat.pdf

Life sciences study design

All studies must disclose on these points even when the disclosure is negative.

Sample size	No sample-size calculation was performed. Repeat 2-3 times in DNA nicking analysis and EMSA analysis. For quantitative data, the experiments were repeated three or four times as it is common practice in the field..
Data exclusions	No data exclusion.
Replication	Values represent the mean ± SD of at least three analyses. And all attempts at replication were successful.
Randomization	This is no relevant to our study. This study focuses on the elucidation of a PT-sensing bacterial defense mechanism. In this study, bioinformatics, structural biology and molecular biology were used to analyze the structure and related functions of SspE protein in PT-sensing bacterial defense system.
Blinding	This is no relevant to our study. This study focuses on the elucidation of a PT-sensing bacterial defense mechanism. In this study, bioinformatics, structural biology and molecular biology were used to analyze the structure and related functions of SspE protein in PT-sensing bacterial defense system.

Reporting for specific materials, systems and methods

We require information from authors about some types of materials, experimental systems and methods used in many studies. Here, indicate whether each material, system or method listed is relevant to your study. If you are not sure if a list item applies to your research, read the appropriate section before selecting a response.

Materials & experimental systems

n/a	Involved in the study
☒	☐ Antibodies
☒	☐ Eukaryotic cell lines
☒	☐ Palaeontology and archaeology
☒	☐ Animals and other organisms
☒	☐ Clinical data
☒	☐ Dual use research of concern

Methods

n/a	Involved in the study
☒	☐ ChIP-seq
☒	☐ Flow cytometry
☒	☐ MRI-based neuroimaging
